# Poor quality care in healthcare settings: an overlooked epidemic

**DOI:** 10.3389/fpubh.2025.1504172

**Published:** 2025-01-24

**Authors:** Augustine Kumah

**Affiliations:** Nyaho Medical Centre, Takoradi, Ghana

**Keywords:** poor-quality care, adverse events, patient safety, patient experience, quality improvement

## Abstract

Adverse events in healthcare settings, including medical errors, healthcare-associated infections (HAIs), and surgical complications, have been a persistent challenge globally, contributing significantly to patient morbidity and mortality. Over the past decade, these events have remained prevalent despite increasing efforts to improve patient safety. This narrative literature review explores the burden of poor-quality care. It examines the trends in adverse events and associated mortalities from 2015 to 2024, highlighting the impact of these events on global health outcomes and identifying potential strategies for reducing their occurrence. Data were collected from various sources such as PubMed, Google Scholar, Scopus, and Ebscohost. The analysis revealed that ~10%−12% of hospitalized patients in high-income countries experienced adverse events annually from 2015 to 2024. Globally, the WHO estimated that 134 million adverse events occurred each year, with ~2.6 million deaths attributed to these events. Adverse events in healthcare settings remain a significant public health challenge, contributing to millions of preventable deaths annually. The persistence of these events highlights systemic issues within healthcare delivery, including inadequate safety protocols, underreporting, and workforce challenges.

## Introduction

The global healthcare landscape has undergone significant transformations in recent decades, with substantial efforts to improve access to healthcare services, particularly in low- and middle-income countries (LMICs). These efforts, encapsulated in global initiatives such as the Millennium Development Goals (MDGs) and the Sustainable Development Goals (SDGs), have primarily focused on increasing the availability of healthcare services to underserved populations (1). However, while access to healthcare has improved in many parts of the world, a critical issue remains largely overlooked: the quality of care provided. Poor quality care, often resulting in adverse health outcomes, has emerged as an epidemic, affecting millions of individuals globally.

Quality care is typically defined by three primary dimensions: safety, effectiveness, and patient-centeredness. Safety refers to the avoidance of harm to patients during the provision of healthcare services. Effectiveness involves delivering healthcare services based on scientific knowledge and evidence-based practices, ensuring patients receive the most appropriate treatment for their condition. Patient-centeredness emphasizes the importance of involving patients in their care, respecting their preferences, and ensuring that their needs and values guide clinical decisions (2). When any of these dimensions are compromised, the quality of care is deemed poor. Unfortunately, healthcare systems in many parts of the world, particularly LMICs, struggle to deliver care consistently, meeting these standards. This has led to a situation where millions of people, even those with access to healthcare, receive care that is suboptimal or outright harmful (3).

### Global burden of poor-quality care

The scale of the problem posed by poor-quality care is staggering. According to the World Health Organization (WHO), ~134 million adverse events occur annually in healthcare settings in LMICs, resulting in 2.6 million deaths each year (4). These figures underscore the magnitude of harm caused by unsafe medical practices, diagnostic errors, and inadequate treatment. In high-income countries, the situation is similarly concerning, with patient safety incidents leading to tens of thousands of deaths annually (5). Poor quality care manifests in various forms, including medical errors, misdiagnoses, medication errors, unsafe surgical procedures, and healthcare-associated infections (HAIs). These issues are not confined to any geographic region but are pervasive across healthcare systems globally (6). The consequences of poor-quality care extend beyond immediate patient harm, contributing to long-term disability, loss of productivity, and increased healthcare costs. Moreover, the fear of receiving poor quality care can deter individuals from seeking necessary medical treatment, further exacerbating health disparities (7).

## Factors contributing to poor quality care

### Healthcare system weaknesses

One of the primary contributors to poor quality care is the weakness of healthcare systems, particularly in LMICs. These weaknesses manifest in various ways, including inadequate infrastructure, insufficient funding, a shortage of trained healthcare professionals, and poor governance. In many LMICs, healthcare facilities are often under-resourced, with limited access to essential medicines, medical equipment, and diagnostic tools (3, 12). These resource constraints hinder the ability of healthcare providers to deliver safe and effective care, leading to substandard treatment and poor patient outcomes. The shortage of healthcare professionals is another significant issue. Many LMICs face a critical shortage of doctors, nurses, and other healthcare workers, resulting in high patient-to-provider ratios and overburdened healthcare staff. This often leads to burnout, fatigue, and errors in patient care (17, 18). Furthermore, the lack of ongoing training and professional development for healthcare workers contributes to a gap in knowledge and skills, further compromising the quality of care provided (4, 8).

### Medical errors and diagnostic failures

Medical errors and diagnostic failures are significant contributors to poor quality care. These errors can occur at various stages of the healthcare process, including diagnosis, treatment, and follow-up. Diagnostic errors, in particular, are a significant concern, with studies suggesting that they affect ~12 million adults in the United States alone each year (19). Misdiagnoses can lead to inappropriate or delayed treatment, resulting in preventable harm or death. Medication errors are another common issue, often arising from incorrect prescribing, dispensing, or administration of drugs. These errors can result in adverse drug reactions, overdose, or underdose, leading to patient harm (5). In many cases, medication errors are preventable through the implementation of better safety protocols, electronic prescribing systems, and enhanced communication among healthcare providers.

### Healthcare-associated infections

Healthcare-associated infections (HAIs) are a significant source of patient harm and are closely linked to poor quality care. HAIs occur when patients acquire infections in a healthcare facility, often due to inadequate infection prevention and control practices. Common HAIs include surgical site infections, catheter-associated urinary tract infections, and ventilator-associated pneumonia (20, 21). The burden of HAIs is exceptionally high in LMICs, where infection prevention and control practices may be insufficient due to resource constraints and lack of training. In these settings, HAIs can lead to increased morbidity and mortality, prolonged hospital stays, and higher healthcare costs (20). Addressing the issue of HAIs requires a multifaceted approach, including improving infection prevention and control practices, ensuring access to clean water and sanitation in healthcare facilities, and promoting hand hygiene among healthcare workers.

### Antimicrobial resistance

Antimicrobial resistance (AMR) is another critical factor in poor-quality care. The overuse and misuse of antimicrobials in healthcare settings have led to the emergence of drug-resistant pathogens, making infections more difficult to treat. AMR is a global health threat with the potential to undermine the effectiveness of essential medical treatments, including surgery, chemotherapy, and organ transplantation (22). In many healthcare settings, particularly in LMICs, the lack of regulation and oversight of antimicroial use has exacerbated the problem of AMR. Patients may receive antibiotics for conditions that do not require them, or they may receive inappropriate doses or durations of treatment. This contributes to the spread of resistant bacteria, which can be transmitted to other patients within healthcare facilities (4, 23). Addressing AMR requires coordinated efforts to promote the rational use of antimicrobials, improve infection control practices, and develop new antimicrobial agents.

### Social determinants of health

The social determinants of health, including poverty, education, and social inequality, also play a significant role in individuals' quality of care. Patients from disadvantaged backgrounds are more likely to experience poor quality care due to factors such as limited access to healthcare services, lower health literacy, and discrimination within the healthcare system (24). In many cases, healthcare facilities serving low-income or marginalized populations may be underfunded and understaffed, leading to substandard care. Additionally, healthcare providers may hold biases or stereotypes that affect their interactions with patients from particular social or ethnic groups, resulting in disparities in the quality of care provided (25). Addressing these social determinants of health is essential to ensuring that all individuals receive high-quality care, regardless of their background or circumstances.

### Governance and policy challenges

Governance and policy issues are critical determinants of healthcare quality. Weak governance, corruption, and inadequate regulation can contribute to poor quality care by allowing unsafe practices to persist, misallocating resources, and failing to enforce standards of care (26–28). One of the significant challenges faced by healthcare systems in LMICs is the outdated or non-existent health policies that fail to address the evolving healthcare needs of these populations (29, 30). In many countries, healthcare policies may be poorly designed or implemented, resulting in gaps in service delivery and disparities in healthcare access and quality (30). Many LMICs operate under policies that have not been revised or are poorly aligned with contemporary healthcare challenges such as antimicrobial resistance, non-communicable diseases, and pandemic preparedness (30). The absence of robust policy frameworks contributes to inefficiencies, inequitable access to care, and persistent quality gaps in healthcare delivery (29, 30). Effective governance is essential to establishing a culture of quality within healthcare systems. This includes setting clear standards and guidelines for healthcare delivery, ensuring accountability among healthcare providers, and promoting transparency in healthcare decision-making (27, 31). Additionally, robust policy frameworks are needed to address issues such as patient safety, AMR, and the social determinants of health.

This narrative review aims to explore the burden of poor-quality care. It examines the trends in adverse events and associated mortalities from 2015 to 2024, highlighting the impact of these events on global health outcomes and identifying potential strategies for reducing their occurrence.

## Methods

This narrative literature review explores the implications of poor-quality care. It examines the trends in adverse events and associated mortalities from 2015 to 2024, highlighting the impact of these events on global health outcomes and identifying potential strategies for reducing their occurrence. Data on poor quality care, adverse events and mortality rates were collected from various sources such as PubMed, Google Scholar, Scopus, and Ebscohost, including reports from the World Health Organization (WHO), the Institute for Healthcare Improvement (IHI), and other relevant studies. The analysis focused on the incidence of adverse events, the resulting deaths, and the contributing factors, with comparisons made across different years to assess trends over time. The data collection process also involved reviewing literature on global health trends to contextualize the quantitative data.

## Discussion

The data on adverse events and the resulting hospital mortalities from 2015 to 2024 paints a stark picture of the challenges healthcare systems face worldwide (Tables 1, 2). Over the past decade, adverse events—defined as harm experienced by patients due to medical care rather than the underlying disease—have persisted at alarming rates. These events include medical errors, hospital-acquired infections, surgical complications, and medication errors. The accompanying mortality data reveals the severe consequences of these events, underscoring the urgent need for systemic improvements in patient safety.

**Table 1:** Adverse events outcomes.

**Year**	**Reporting agency**	**Adverse events outcomes (measures and sources)**
2015	WHO, U.S. Reports	12 million adverse events in the U.S.; ~10% of hospitalized patients in high-income countries were affected. (Source: WHO, Makary and Daniel, 2016)
2016	WHO	134 million adverse events globally, primarily in LMICs. (Source: WHO, 2019)
2017	WHO	134 million adverse events globally. (Source: WHO, 2029)
2018	OECD	15% of all hospital expenditures were linked to adverse events. (Source: OECD Report, 2018)
2019	NHS	1.8 million adverse events were reported in English hospitals. (Source: NHS, 2019)
2020	WHO	Adverse events increased due to COVID-19, with 134 million annually. (Source: WHO, 2020)
2021	WHO	10%−12% of patients in high-income countries experienced adverse events. (Source: WHO, 2024)
2022	WHO	10% of patients in high-income countries experienced adverse events. (Source: WHO, 2024)
2023	WHO	Continued high rates of adverse events were reported. (Source: WHO, 2024)
2024	WHO	Projected 134 million adverse events globally. (Source: WHO, 2024)

**Table 2:** Mortality from adverse events.

**Year**	**Reporting agency**	**Mortality from adverse events (measures and sources)**
2015	WHO, U.S. Reports	Estimated 250,000 deaths in the U.S. due to medical errors. (Source: Makary & Daniel, 2016)
2016	WHO	Approximately 2.6 million deaths worldwide were attributed to adverse events. (Source: WHO, 2018)
2017	WHO	Approximately 2.6 million deaths worldwide were due to adverse events. (Source: WHO, 2018)
2018	WHO	Around 2.6 million deaths globally from adverse events. (Source: WHO, 2018)
2019	NHS	11,000 severe harms or deaths in the NHS system. (Source: NHS, 2019)
2020	WHO	Continued estimate of 2.6 million deaths globally. (Source: WHO, 2020)
2021	WHO	Over 2.5 million deaths globally, with an increase due to pandemic strain. (Source: WHO, 2024)
2022	WHO	2.6 million deaths globally from adverse events. (Source: WHO, 2024)
2023	WHO	Estimated 2.5-2.6 million deaths globally post-pandemic. (Source: WHO, 2024)
2024	WHO	Projected 2.6 million deaths globally unless safety measures are improved. (Source: WHO, 2024)

## Trends in adverse events and mortalities

### Persistent high rates of adverse events

In the decade from 2015 to 2024, the incidence of adverse hospital events remained persistently high (Tables 1, 2). The data indicates that ~10%−12% of hospitalized patients in high-income countries experienced some form of adverse event each year (5, 32). This percentage translates to millions of patients worldwide being affected by preventable harm during their hospital stay. For instance, in 2015, an estimated 12 million adverse events occurred in the United States alone, affecting roughly 10% of hospitalized patients (5). Globally, the WHO estimated that 134 million adverse events occurred annually, particularly in low- and middle-income countries (LMICs), where healthcare systems often struggle with resource constraints and less rigorous safety protocols (8). These figures remained relatively consistent throughout the subsequent years, with no significant reduction in adverse event rates, even as awareness and efforts to improve patient safety increased.

### Adverse events and healthcare costs

One notable finding is the significant economic impact of adverse events. In 2018, the WHO reported that 15% of all hospital expenditures in OECD countries were linked to adverse events. These adverse events also contribute to extended hospital stays, with patients experiencing an average of 4–7 additional days of hospitalization, depending on the event's severity (13, 14, 33, 34). Economically, the global cost of adverse events is staggering, with unsafe medical practices accounting for an estimated $42 billion annually (32, 33). Such figures underscore the urgent need for targeted interventions to mitigate healthcare systems' financial and operational burdens. This statistic highlights the substantial financial burden that adverse events place on healthcare systems. Resources that could otherwise be directed toward improving patient care and outcomes are instead used to manage the fallout from preventable harm. Moreover, the financial implications extend beyond direct healthcare costs. Adverse events often result in prolonged hospital stays, additional treatments, and legal expenses, all contributing to the overall economic burden. The cumulative effect of these costs underscores the importance of investing in preventive measures and safety protocols to reduce the incidence of adverse events.

### Mortality rates from adverse events

The data on mortality rates associated with adverse events is perhaps the most alarming aspect of these findings. Each year, millions of deaths worldwide are attributed to adverse events, with little variation in mortality rates over the decade (35–37). In 2016, the WHO estimated that 2.6 million deaths globally were a direct result of adverse events, which remained consistent through 2024 (4, 13, 16, 34). In high-income countries like the United States, medical errors alone were estimated to cause around 250,000 deaths annually, making them the third leading cause of death in the country (5, 33). This statistic is particularly troubling as it highlights that many of these deaths are preventable. The persistent nature of these mortality rates suggests that systemic issues within healthcare systems are not adequately addressed.

### The impact of COVID-19 on adverse events and mortalities

The COVID-19 pandemic, which began in 2020, profoundly impacted healthcare systems worldwide, exacerbating adverse events. The pandemic placed unprecedented strain on hospitals, leading to increased rates of hospital-acquired infections, medication errors, and other adverse events as healthcare providers struggled to manage the overwhelming number of patients. The data from 2020 reflects this impact, with WHO reports indicating an increase in adverse events due to the challenges posed by the pandemic (13, 14, 16). Although the exact numbers are difficult to quantify, the pandemic undoubtedly worsened the patient safety crisis, particularly in regions where healthcare systems were already under-resourced (Tables 1, 2).

### Projected trends and future implications

Looking ahead to 2024, the data projects that without significant improvements in patient safety measures, the rates of adverse events and associated mortalities will remain alarmingly high. The WHO continues to project ~134 million adverse events annually, with 2.6 million deaths resulting from these events (13, 16). These projections emphasize the need for comprehensive and sustained efforts to improve patient safety across all healthcare settings.

## Factors contributing to persistent poor-quality care

### Systemic issues in healthcare delivery

The persistence of high rates of adverse events and related mortalities suggests that systemic issues within healthcare delivery are a major contributing factor. These issues include inadequate staffing levels, insufficient training, poor communication among healthcare providers, and a lack of robust safety protocols. In many healthcare settings, particularly LMICs, the resources necessary to implement adequate safety measures are limited (3, 12, 38, 39). Additionally, the complexity of modern healthcare, with its reliance on advanced technology and multidisciplinary teams, increases the potential for errors. In high-income countries, while technology and protocols are more advanced, the sheer volume of patients and the complexity of their care can lead to breakdowns in communication and coordination, resulting in adverse events.

### Underreporting and lack of accountability

Another factor contributing to the ongoing issue of adverse events is underreporting. Many adverse events go unreported due to fear of blame, lack of awareness, or inadequate reporting systems (28, 29, 40). This lack of transparency prevents healthcare providers and institutions from learning from these events and implementing necessary changes to prevent future occurrences (9, 29, 40). Moreover, the lack of accountability within some healthcare systems means that when adverse events occur, there are often few consequences for those responsible. This lack of accountability can perpetuate a culture of complacency, where the urgency to improve patient safety is diminished.

### The role of healthcare culture

The culture within healthcare organizations plays a significant role in perpetuating or mitigating adverse events. In environments with a blame culture, healthcare providers may be reluctant to report errors, hindering efforts to address systemic issues (38, 40). Conversely, organizations that promote a safety culture—where staff are encouraged to report errors and near misses without fear of retribution—are more likely to see improvements in patient safety (41–43). Creating a safety culture requires leadership commitment, ongoing education and training, and implementing systems that support open communication and continuous improvement. Without these elements, efforts to reduce adverse events and improve patient outcomes will likely fall short.

## Recommendations for reducing adverse events and mortalities

### Strengthening safety protocols and systems

One of the most effective ways to reduce adverse events and associated mortalities is to strengthen safety protocols and systems within healthcare settings. This includes implementing standardized procedures for everyday medical tasks, ensuring that all staff are adequately trained, and using technology to reduce the potential for human error (15, 42). For example, electronic health records (EHRs) can help prevent medication errors by providing healthcare providers with accurate and up-to-date information about patients' medications and allergies. Similarly, surgical safety checklists have been shown to reduce the incidence of surgical complications and improve patient outcomes (44).

### Enhancing reporting and accountability mechanisms

Improving reporting and accountability mechanisms is crucial for addressing the issue of underreporting and fostering a culture of safety. Healthcare organizations should implement robust incident reporting systems that encourage staff to report adverse events and near misses without fear of blame. These systems should be used not only to track and analyze adverse events but also to identify patterns and underlying causes, leading to targeted interventions (9–11, 39, 45). Additionally, healthcare providers and institutions should be held accountable for maintaining patient safety standards. This could involve regular audits of safety practices and penalties for organizations that fail to comply with established safety protocols. At the same time, it is essential to recognize and reward organizations that demonstrate excellence in patient safety, encouraging others to follow suit.

### Investing in workforce training and development

Ongoing training and development for healthcare professionals are essential for reducing adverse events and improving patient safety. This includes initial training for new staff and continuous professional development opportunities that keep healthcare providers up to date with the latest safety protocols and best practices (15, 46). Investing in workforce training also addresses burnout and fatigue, which can contribute to errors. Ensuring that healthcare providers have manageable workloads and access to mental health support can help reduce the risk of errors and improve overall patient safety (15, 42).

### Fostering a culture of safety

Creating a safety culture within healthcare organizations is essential in reducing adverse events and associated mortalities. This involves leadership commitment to patient safety, clear communication of safety priorities, and establishing systems that support continuous improvement (38–40). Healthcare leaders should prioritize patient safety in all aspects of care delivery, from policy development to daily operations. This includes fostering an environment where staff feel empowered to speak up about safety concerns and where errors are viewed as opportunities for learning rather than reasons for punishment (41).

### Leveraging technology and innovation

Finally, leveraging technology and innovation can significantly reduce adverse events and improve patient outcomes. Advances in health information technology, such as EHRs and clinical decision support systems, can help reduce errors and improve the accuracy of diagnosis and treatment (47). Additionally, emerging technologies such as artificial intelligence and machine learning hold promise for predicting and preventing adverse events before they occur. Investment in research and development is also crucial for driving innovation in patient safety. By supporting the development of new tools, technologies, and approaches, healthcare organizations can stay ahead of emerging risks and continually improve the quality of care they provide (45).

## Conclusion

Poor quality care is an overlooked epidemic that significantly threatens global health. The consequences of poor-quality care are far-reaching, affecting health outcomes, mortality, economic stability, and patient trust. Addressing this epidemic requires a comprehensive and multifaceted approach, with efforts to strengthen healthcare systems, enhance patient safety, combat antimicrobial resistance, and address the social determinants of health. To reduce adverse events and improve patient outcomes, healthcare organizations must strengthen safety protocols, enhance reporting and accountability mechanisms, invest in workforce training, foster a safety culture, and leverage technology and innovation. By taking these steps, healthcare providers can protect patients from harm and ensure that hospitals are places of healing rather than sources of preventable injury or death. Policy and governance reforms and leveraging technology and innovation are essential to improving healthcare quality and ensuring all individuals receive safe, effective, patient-centered care. By recognizing and addressing the issue of poor-quality care, we can work toward a future where healthcare is truly a force for good, improving health outcomes and reducing health disparities for all.

## References

[1] World Health Organization. Health in 2015: From MDGs, Millennium Development Goals to SDGs, Sustainable Development Goals. Geneva: World Health Organisation. (2015).

[2] Abid MH Kumah A Newera A Hafez P. Patient-centered healthcare: from patient experience to human experience. Glob J Qual Saf Healthc. (2024) 7:144–8. 10.36401/JQSH-24-X2 39534234 PMC11554389

[3] Kruk ME Ling EJ Bitton A Cammett M Cavanaugh K Chopra M. Building resilient health systems: a proposal for a resilience index. BMJ. (2017) 357:j2323. 10.1136/bmj.j2323 28536191

[4] WHO. WHO | The Third WHO Global Patient Safety Challenge: Medication Without Harm. Geneva: WHO (2019).

[5] Makary MA Daniel M. Medical error-the third leading cause of death in the US. BMJ. (2016) 353:i2139. 10.1136/bmj.i2139 27143499

[6] Jha AK Larizgoitia I Audera-Lopez C Prasopa-Plaizier N Waters H Bates DW. Patient safety: global action on patient safety - report by the director-general (Vol. 22). Geneva: World Health Organization (2018).

[7] Vincent C Amalberti R. (eds.). Progress and challenges for patient safety. In: *Safer Healthcare*. Springer (2016). p. 3–18. 10.1007/978-3-319-25559-0_1

[8] WHO. WHO | Health Systems Financing: The Path to Universal Coverage. Geneva: WHO. (2016). 10.2471/BLT.10.078741 PMC2878164 20539847

[9] Panagioti M Khan K Keers RN Abuzour A Phipps D Kontopantelis E. Prevalence, severity, and nature of preventable patient harm across medical care settings: Systematic review and meta-analysis. BMJ. (2019) 366:l4185. 10.1136/bmj.l4185 31315828 PMC6939648

[10] World Health Organization. Global Action on Patient Safety. WHO Director General report to 72nd World Health Assembly. Geneva (2019).

[11] World Health Organization. Patient Safety Incident Reporting and Learning Systems. Geneva (2020).

[12] Kruk ME Gage AD Arsenault C Jordan K Leslie HH Roder-DeWan S. High-quality health systems in the sustainable development goals era: time for a revolution. Lancet Glob Health. (2018) 6:e1196–252. 10.1016/S2214-109X(18)30386-3 30196093 PMC7734391

[13] World Health Organization (WHO). Global Patient Safety Report 2024. Geneva (2024). Available at: https://iris.who.int/

[14] Dhingra-Kumar N Brusaferro S Arnoldo L. Patient safety in the world. In: Ricciardi W, Flahault A, Manca MG, Melegaro A, editors. Textbook of Patient Safety and Clinical Risk Management. Springer (2021). pp. 89–105. 10.1007/978-3-030-59403-9_8

[15] World Health Organization. Global Patient Safety Action Plan 2021–2030: Towards Eliminating Avoidable Harm e Health Care. Geneva (2021).

[16] World Health Organization (WHO). Monitoring Health for the SDGs, Sustainable Development Goals. Geneva (2024).

[17] Campbell CA Horvath AR. Harmonization of critical result management in laboratory medicine. Clinica Chimica Acta. (2014) 432:135–47. 10.1016/j.cca.2013.11.004 24246790

[18] Campbell SM Roland MO Buetow SA. Defining quality of care. Soc Sci Med. (2000) 51:1611–25. 10.1016/S0277-9536(00)00057-5 11072882

[19] Singh H Schiff GD Graber ML Onakpoya I Thompson MJ. The global burden of diagnostic errors in primary care. BMJ Qual Saf . (2017) 26:5401. 10.1136/bmjqs-2016-005401 27530239 PMC5502242

[20] Nejad SB Allegranzi B Syed S Ellis B Pittet D. Health-care-associated infection in Africa: a systematic review. Bull World Health Organ. (2011) 89:757–65. 10.2471/BLT.11.088179 22084514 PMC3209981

[21] Allegranzi B. Regional differences impacting infection control practices. Int J Infect Dis. (2012) 16:E3–4. 10.1016/j.ijid.2012.05.013

[22] O'Neill J. The Review on Antimicrobial Resistance Tackling drug-Resistant Infections Globally: Final Report and Recommendations. London: Review on Antimicrobial Resistance (2014).

[23] Laxminarayan R Duse A Wattal C Zaidi AK Wertheim HF Sumpradit N. Antibiotic resistance-the need for global solutions. Lancet Infect Dis. (2013) 13:1057–98. 10.1016/S1473-3099(13)70318-9 24252483

[24] Braveman P Egerter S Williams DR. The social determinants of health: coming of age. Annu Rev Public Health. (2011) 32:381–98. 10.1146/annurev-publhealth-031210-101218 21091195

[25] Burgess D Van Ryn M Dovidio J Saha S. Reducing racial bias among health care providers: lessons from social-cognitive psychology. J Gen Intern Med. (2007) 22:882–7. 10.1007/s11606-007-0160-1 17503111 PMC2219858

[26] Frenk J Moon S. Governance challenges in global health. N Engl J Med. (2013) 368:9339. 10.1056/NEJMra1109339 23465103

[27] Lane J Andrews G Orange E Brezak A Tanna G Lebese L. Strengthening health policy development and management systems in low- and middle- income countries: South Africa's approach. Health Policy Open. (2020) 1:100010. 10.1016/j.hpopen.2020.100010 37383321 PMC10297791

[28] Hudson B Hunter D Peckham S. Policy failure and the policy-implementation gap: can policy support programs help? Policy Design Pract. (2019) 2:1–14. 10.1080/25741292.2018.1540378

[29] Yanful B Kirubarajan A Bhatia D Mishra S Allin S Di Ruggiero E. Quality of care in the context of universal health coverage: a scoping review. Health Res Policy Syst. (2023) 21:21. 10.1186/s12961-022-00957-5 36959608 PMC10035485

[30] George J Jack S Gauld R Colbourn T Stokes T. Impact of health system governance on healthcare quality in low-income and middle-income countries: a scoping review. BMJ Open. (2023) 13:73669. 10.1136/bmjopen-2023-073669 38081664 PMC10729209

[31] WHO. WHO Consolidated Guideline on Self-Care Interventions for Health. Geneva (2019). 31334932

[32] World Health Organization. Health Care-Associated Infections Fact Sheet. Geneva: World health Organization (2015).

[33] Auraaen A Slawomirski L Klazinga N. The economics of patient safety in primary and ambulatory care: FLYING BLIND. OECD Health Working Papers. (2018) 2018:106.

[34] Venkatesan L. Patient safety challenge: medication without harm. Nurs J India. (2022) CXIIIL:8. 10.48029/NJI.2022.CXIII507

[35] Lunevicius R Haagsma JA. Incidence and mortality from adverse effects of medical treatment in the UK, 1990-2013: Levels, trends, patterns and comparisons. Int J Qual Health Care. (2018) 30:558–64. 10.1093/intqhc/mzy068 29659841 PMC6094799

[36] Haukland EC Mevik K Von Plessen C Nieder C Vonen B. Contribution of adverse events to death of hospitalised patients. BMJ Open Qual. (2019) 8:e000377. 10.1136/bmjoq-2018-000377 30997413 PMC6440591

[37] Nauman J Soteriades ES Hashim MJ Govender R Al Darmaki RS Al Falasi RJ. Global incidence and mortality trends due to adverse effects of medical treatment, 1990–2017: a systematic analysis from the global burden of diseases, injuries and risk factors study. Cureus. (2020) 12:e7265. 10.7759/cureus.7265 32195071 PMC7075477

[38] Forkuo-Minka AO Kumah A Asomaning AY. Improving patient safety: learning from reported hospital-acquired pressure ulcers. Glob J Qual Saf Healthc. (2024) 7:15–21. 10.36401/JQSH-23-25 38406657 PMC10887484

[39] Kumah A Nwogu CN Issah AR Obot E Kanamitie DT Sifa JS. Cause-and-effect (Fishbone) diagram: a tool for generating and organizing quality improvement ideas. Glob J Qual Saf Healthc. (2024) 7:85–7. 10.36401/JQSH-23-42 38725884 PMC11077513

[40] Kumah A Zon J Obot E Yaw TK Nketsiah E Bobie SA. Using incident reporting systems to improve patient safety and quality of care. Glob J Qual Saf Healthc. (2024) 7:228–31. 10.36401/JQSH-23-39 39534243 PMC11554398

[41] Vincent C Amalberti R. Safer Healthcare: Strategies for the Real World. Cham, CH: Springer (2016). 10.1007/978-3-319-25559-0 29465922

[42] Hibbert PD Stewart S Wiles LK Braithwaite J Runciman WB Thomas MJW. Improving patient safety governance and systems through learning from successes and failures: qualitative surveys and interviews with international experts. Int J Qual Healthc. (2023) 35:0. 10.1093/intqhc/mzad088 37978851 PMC10656601

[43] Gandhi TK Kaplan GS Leape L Berwick DM Edgman-Levitan S Edmondson A. Transforming concepts in patient safety: a progress report. BMJ Qual Saf . (2018) 27:1019–26. 10.1136/bmjqs-2017-007756 30018115 PMC6288701

[44] Cadman V. Use of the WHO surgical safety checklist in low and middle income countries: a review of the literature. J Perioper Pract. (2018) 28:334–8. 10.1177/1750458918776551 29737922

[45] Kumah A Nutakor HS Issah AR Obot E Antoinette Aidoo L Selase Sifa J. Achieving sustainability of quality improvement projects. Glob J Qual Saf Healthc. (2024). 10.36401/JQSH-23-48 PMC11808851 39935715

[46] Kumah A. Incorporating healthcare quality improvement into health professional training curriculum in Ghana: insights and perspectives. SSRN. (2024). 10.2139/ssrn.5038370

[47] Jha AK Larizgoitia I Audera-Lopez C Prasopa-Plaizier N Waters H Bates DW. The global burden of unsafe medical care: Analytic modelling of observational studies. BMJ Qual Saf . (2013) 22:1748. 10.1136/bmjqs-2012-001748 24048616

